# A pilot feasibility study of a tablet-based virtual community application with shared avatars for promoting health behavior change in older adult care facilities

**DOI:** 10.3389/fdgth.2026.1757054

**Published:** 2026-05-12

**Authors:** Kenji Nakamura, Keiichi Yamazaki, Akiko Yamazaki, Yoshiaki Ohyama

**Affiliations:** 1 Center for Mathematics and Data Science, Gunma University, Gunma, Japan; 2 Innovative Medical Research Center, Gunma University Hospital, Gunma, Japan; 3 Graduate School of Humanities and Social Sciences, Saitama University, Saitama, Japan; 4 Faculty of Media Studies, Tokyo University of Technology, Tokyo, Japan

**Keywords:** avatars, behavior change, blockchain, medication management, older adults, virtual space, wearable devices

## Abstract

**Background:**

Maintaining exercise and medication habits is crucial for older adults, but conventional reminder-based digital interventions often produce only transient effects.

**Methods:**

We conducted a mixed-methods pilot feasibility study comparing a reminder-only intervention (Experiment 1, *N* = 10) with a healthcare community app combining reminders and avatar-based virtual social interaction (Experiment 2, *N* = 10) in older adult care facilities over 4-week baseline and 4-week intervention periods. Primary outcomes were daily step counts and medication adherence.

**Results:**

The Reminder group showed a transient increase in step count in Week 1 (+16%) that declined to below baseline by Week 4 (−10.3%). In contrast, the Reminder + Support group maintained substantial step count improvements throughout the intervention (Week 4: +58.4%), with statistically significant differences at all time points. Medication adherence showed similar trends. A between-group comparison (Mann-Whitney U = 11.0, *p* < 0.05) supported greater efficacy of the Reminder + Support intervention. Qualitative data confirmed higher satisfaction and stronger social motivation in the Reminder + Support group.

**Conclusion:**

These preliminary findings suggest that integrating avatar-based social interaction with reminder functions may support more sustained health behavior change in older adults than reminders alone, warranting confirmation in larger randomized trials.

## 1 Introduction

**Figure 1:**
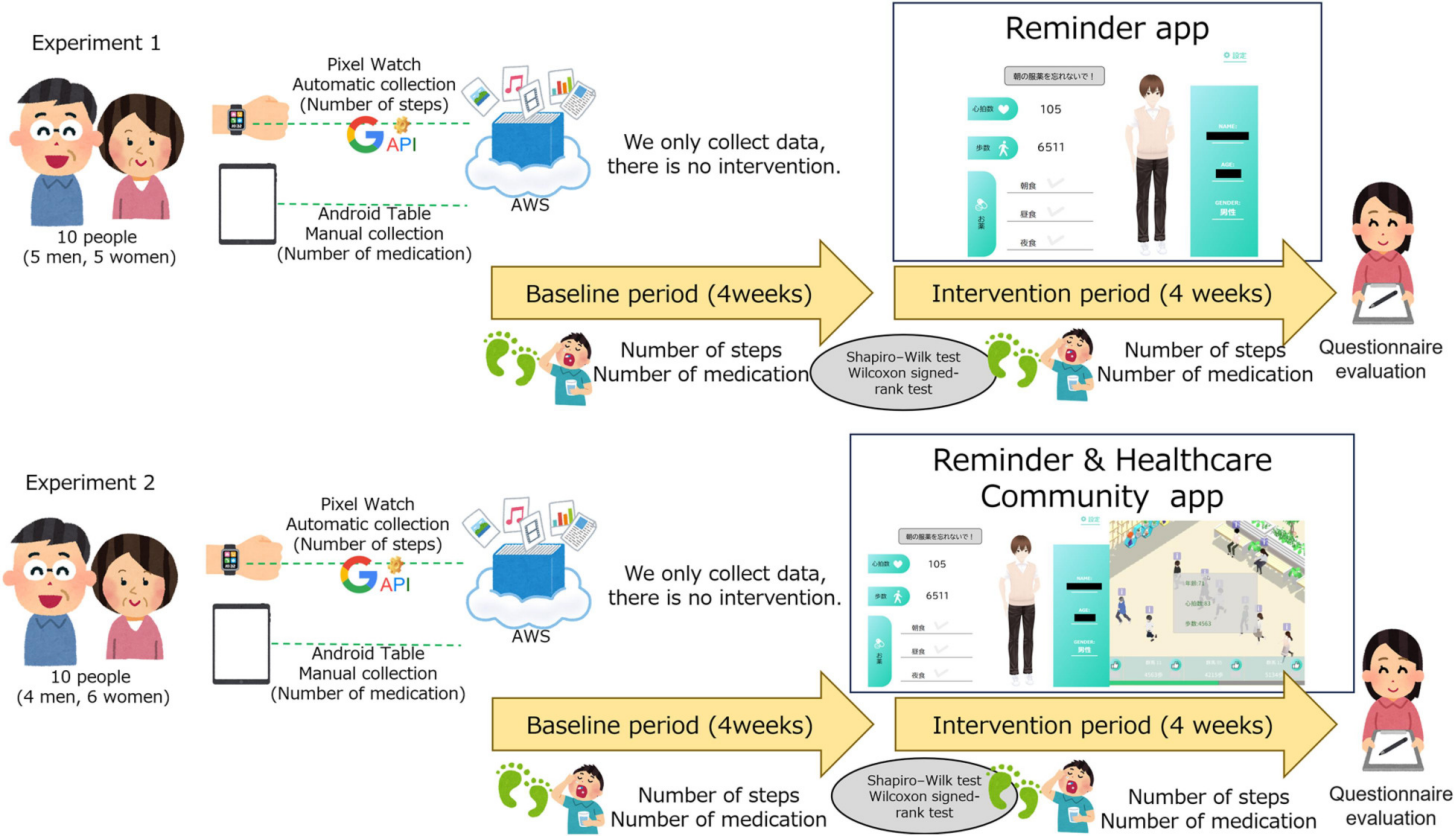
Overall study design.

As the population ages, the number of older adults (65 years and older) worldwide is projected to reach approximately two billion by 2050, nearly double the current figure (1). Although the average life expectancy is increasing, gains in healthy life expectancy have not kept pace; consequently, older adults face concerns of declining quality of life due to multiple chronic illnesses and increased care needs (2). Under these circumstances, ensuring daily exercise habits and medication adherence are among the most important challenges in maintaining older adults’ health. Regular physical activity is indispensable for preventing chronic diseases and maintaining cognitive function. National guidelines recommend that older adults engage in at least 150 min of moderate-intensity exercise per week. However, most older adults do not meet this standard; the United States (U.S.) statistics indicate that only approximately 13.9% of people aged 65 years or above achieve the recommended amounts of both aerobic exercise and muscle-strengthening activities (3). Moreover, medication adherence among older patients is low; a World Health Organization report states that in developed countries, only approximately 50% of patients with chronic diseases take their medications as prescribed (4). Age-related cognitive decline and polypharmacy make it difficult for older adults to comply with medication regimens, and poor adherence directly leads to treatment failure, disease progression, increased rehospitalization, higher healthcare costs, and mortality risks. Thus, while the continuation of exercise and medication routines is essential for healthy longevity among older adults, sustaining these behaviors presents several challenges.

Recently, digital health interventions that leverage technology have attracted attention as a means of supporting older adults. Specifically, smartphone health management applications, wearable activity trackers, electronic medication alert devices, and remote monitoring systems have been developed and applied to promote physical activity and manage medication adherence among older adults (5). Several digital interventions have demonstrated their effectiveness; for example, Yoon et al. (6) reported that providing a smartphone medication management app (ADHERE-App) to patients with atrial fibrillation significantly improved adherence to the anticoagulant edoxaban, with six-month follow-up indicating improved medication continuation rates. However, many older adults are unfamiliar with smartphones and similar devices, creating challenges in ensuring that digital interventions are both adopted and sustained over time. The ADHERE-App trial was limited to participants with high digital literacy, indicating that its generalizability to a broader older adult population is limited. Moreover, the effectiveness of digital health interventions for older adults varies depending on the intervention content, and some simple interventions appear insufficient to achieve meaningful behavioral changes. Jeon et al.'s (7) meta-analysis on medication adherence interventions among older patients with chronic diseases revealed that interventions, including digital technology, had a moderate average effect size, but those combining face-to-face individualized counseling with practical behavior-change strategies were more effective. However, interventions comprising only remote support—such as information provision or phone calls—showed no significant effect, suggesting that interactive and actively engaging support strategies are indispensable for older adults. Similarly, a survey by Nguyen et al. (8) identified several challenges specific to older adults: Diminished responsiveness owing to frequent notifications (so-called “alert fatigue”), low health literacy, lack of social support from family or community, and difficulty in motivating behavior change. Thus, interventions using digital technology must incorporate ways to overcome these psychosocial barriers to sustain behavioral changes in older adults. Additionally, some reports suggest that incorporating appealing motivational strategies tailored to each individual is important for enhancing the long-term effectiveness and generalizability of digital health interventions (9).

Considering this background, the present study proposes a novel approach to promote the maintenance of exercise and medication habits among older adults using a community healthcare application. This application combines a virtual-space avatar-sharing system with wearable-device activity tracking. Specifically, it uses avatars that mirror each participant's real-world activities (such as performing an activity and taking medication) in real time, creating a community environment in which participants can visually and intuitively share their health behaviors. The system is designed so that participants can gauge their own activity levels by observing others’ avatar movements, and through simple virtual interactions—such as clicking on “like” or leaving comments—they receive social motivation in the form of peer approval and encouragement. Additionally, by anonymously recording and sharing each participant's data (e.g., step counts and medication history) on a blockchain, the system prevents data tampering and ensures high data integrity. It also protects privacy and streamlines information sharing among participants, caregivers, and healthcare providers. This study aims to empirically verify the effectiveness of this healthcare community app in supporting the maintenance of health behaviors among older adults. An intervention experiment using the system was conducted in an older adult facility, comparing a basic reminder-only intervention with an intervention delivered through the community app, which included avatar sharing and a “like” feature. This paper reports the results of the experiment and discusses the effectiveness of the approach and its potential applications.

## 2 Related work

Various intervention trials using different approaches have been reported in prior research on medication adherence and exercise promotion among older adults. We focus on prior studies in four areas: leveraging social motivation, interventions using avatars, the psychological effects of approval buttons (e.g., the “like” feature), and the use of blockchain technology to ensure anonymity.

Social support from peers or family plays an important role in promoting behavioral change among older adults. Social encouragement and relationships involving competition or cooperation tend to lead to sustained motivation, and the presence of others can enhance an individual's performance. Therefore, previous studies have attempted to involve family members or partners in interventions to improve medication adherence. For example, Kessler et al. (10) reported that in a trial among patients with chronic diseases, an intervention combining support from an assigned medication partner with automated alert notifications improved medication adherence rates, whereas partner support alone did not produce a significant improvement. This suggests that simply assigning a peer supporter is not sufficient; it may be necessary to integrate such support with technological reminders or provide a mechanism for supporters to engage effectively. Furthermore, lack of social support from the community or family can be a barrier to sustaining healthy behaviors in older adults (8). Therefore, incorporating elements of social interaction into digital interventions is expected to help maintain motivation among older adults. Theoretically, as the social learning theory indicates, learning is facilitated by observing and imitating others; therefore, peer stimuli and role models can serve as powerful drivers of behavioral change (11).

In the digital realm, approaches that incorporate social factors through avatars (as virtual embodiments) and virtual environments have gained increasing attention. Prior studies involving older adults have demonstrated the usefulness of interventions that employ these tools. For example, Cook and Winkler's (5) study that examined older adults’ adaptability to a virtual-world platform found that they were generally receptive to interaction and engagement via avatars, suggesting that virtual worlds have the foundation required for acceptability among older adults. Additionally, Drazich et al. (12) conducted a randomized pilot trial (the MOTIVE study) of an exercise intervention using immersive virtual reality (VR) and reported that the VR-based exercise program was well accepted by older participants and showed promise for encouraging physical activity. Tammy Lin and Wu (13), in their research on avatar design and presentation, demonstrated that using a youthful-looking avatar in VR can reduce an older adult's perceived exertion during exercise, suggesting that having a younger avatar embody the participant may provide psychological benefits for exercise adherence. According to Da Silva et al. (14), adherence was higher when virtual bowling exercises were performed with peers than when they were performed alone. This suggests that the sense of interpersonal connection fostered through virtual spaces can have a positive impact on adherence (14). Furthermore, in an effort to engage older adults in virtual group exercises, Elizabeth et al. (15) developed an avatar-based virtual exercise class and demonstrated that older adults could enjoy participating in physical activity (15). Additionally, not only humanoid avatars but also entities such as digital pets can provide psychological support for older **adults**. Pilot studies by Chi et al. (16) provided older adults with a digital pet avatar as a daily companion to enhance emotional reassurance and motivate active physical activity, and participants reportedly responded positively. Thus, previous research suggests that interventions utilizing avatars and virtual environments are acceptable for older adults and can be effective in promoting physical activity and behavioral change. Gaining a sense of embodiment in full-body ownership through an avatar can influence users’ cognition and behavior (17). Taken together, these findings indicate that avatar-mediated experiences can serve as a foundation for changing user psychology and behavior, thereby supporting the potential effectiveness of the avatar-sharing approach used in this study. More broadly, recent work on immersive technologies in older adults further supports the relevance of this trajectory. A systematic mapping review reported that VR/AR applications for older adults span multiple quality-of-life domains, including physical, cognitive, psychological, and social domains, while also emphasizing the importance of clearly characterizing the technologies used (18). In addition, a participatory design study of collaborative AR activities in long-term care showed that AR experiences can be tailored to older adults’ physical and cognitive needs and may support social interaction when carefully designed for this population (19). Furthermore, a feasibility study of active augmented reality games in older adults suggested that AR-based interventions may be an acceptable and behaviorally meaningful modality in this population (20).

The “like” button (approval button) commonly used on social media provides a simple way to receive positive feedback from others and is a tool that can influence users’ psychology. According to prior research, receiving “likes” online activates brain regions associated with the reward system and, as a form of positive social evaluation, may influence the recipient's motivation to act (21). Such social feedback can fulfill the need for approval and strengthen self-efficacy, acting as a positive reinforcement for behavior. On popular social networking platforms, users whose posts receive numerous “likes” or positive comments tend to show increased subsequent activity (21), suggesting that praise itself can serve as an incentive for sustained engagement. In supporting health behaviors among older adults, even small words of praise from peers (e.g., a comment like “Your step count has really increased—great job!”) are believed to help maintain participants’ motivation. A “like” that can be easily sent via an app may serve as a means of fostering positive social connections in environments where in-person interaction is limited. In the app developed for this study, the “like” function is intended to leverage the positive reinforcement effect of such social approval, and as suggested by prior work, it is expected to be a factor that increases participants’ motivation for continued use. In addition, the exploratory correlation observed between Likes received and step-count improvement provides preliminary support for a possible dose–response relationship between social engagement in the virtual environment and behavioral change. This finding is consistent with the interpretation that positive peer feedback may have reinforced motivation for physical activity within the avatar-mediated setting. However, given the pilot-sized sample and the observational nature of this within-group analysis, the result should be interpreted as supportive rather than confirmatory evidence, and not as definitive causal proof. Future studies with larger samples and prospectively defined engagement metrics will be needed to test this mechanism more rigorously.

Finally, we review previous research on the application of blockchain technology in healthcare. Blockchain—a form of distributed ledger technology—has gained attention as a platform for sharing medical and health data because once recorded, the data cannot be practically altered, ensuring high reliability and transparency. Wan et al. (22) proposed a framework called “MedAlert” to address the issue of “alert fatigue” caused by excessive warnings in clinical settings. In this system, patients and providers share low-level alert information on a blockchain and make decisions collaboratively. The study employed a private blockchain using Hyperledger Fabric to ensure alert data immutability and privacy, and it included a mechanism that allowed patients to view alert information and participate in deciding which alerts to act upon. Similarly, Vazirani et al. (23) reported a case of medical innovation that combined blockchain with brainstorming techniques, noting the potential of decentralized technologies in healthcare research. However, some participants expressed concerns that using this technology may be difficult for those unfamiliar with it, highlighting the challenges older users face when directly interacting with and understanding advanced technologies such as blockchain. A pilot study by Nakamura et al. (24) experimentally introduced a mutual-aid system in which older adults used wearable devices and blockchain to share and verify each other's health data. Avatars were not employed; rather, participants viewed another's health data and provided mutual support, leading to positive changes in step counts and activity levels. This suggests that integrating anonymous, secure blockchain-based data sharing with peer support may effectively promote behavioral changes among older adults. Building on these prior findings, we propose that combining social motivational elements with a highly reliable digital data-sharing platform can offer an innovative solution to the challenges of sustaining health behaviors among older adults. To date, no comprehensive intervention that integrates avatar-based visualization with the reliability assurance of blockchain has been reported; therefore, this study and its approach are highly novel in the context of health behavior support for older adults.

## 3 Methods

### 3.1 Research design

This study employed an exploratory mixed-methods pilot feasibility design to evaluate the implementation, usability, and preliminary behavioral outcomes of a community healthcare application among older adults residing in care facilities in Gunma Prefecture. The reporting of this study adheres to the CONSORT-EHEALTH (V 1.6.1) guidelines for trials of electronic and mobile health applications. As this study evaluates behavioral interventions using independent pilot groups rather than a fully randomized allocation process, we also followed the Transparent Reporting of Evaluations with Non-randomized Designs (TREND) statement to guide the reporting structure. In accordance with TREND recommendations, we explicitly describe the theoretical framework (Social Learning Theory), the assignment procedures between the reminder-only and avatar-supported conditions, and the comparability of baseline characteristics between groups. Detailed parameters, including the mode of delivery (mobile tablet and wearable device), user literacy requirements, and the specific version of the healthcare community app (V 1.6.1), are described to facilitate future replication and theory-building. Each experiment used a separate group of participants and consisted of a four-week baseline observation period followed by a four-week intervention period. Prospective participants were fully informed about the study's purpose, methods, anticipated risks and benefits, and privacy protection policies, and written informed consent, as well as an emergency contact's details, were obtained from each participant. Eligible participants were aged 50 years or older and were regularly taking prescription medications. In addition to these criteria, digital literacy was assessed using a modified Mobile Device Proficiency Questionnaire (MDPQ-16). Participants were required to have a baseline score of ≥20, indicating a basic ability to operate touch screens and mobile applications. This criterion was introduced to ensure that participants could meaningfully interact with the digital intervention and to minimize potential confounding effects arising from difficulties in operating the system. Exclusion criteria eliminated those with severe cognitive impairment or illnesses preventing the use or operation of wearable devices. Finally, 10 older adults (five men, five women; mean age 66.2 ± 5.2 years) participated in Experiment 1 (Reminder group) and 10 (four men, six women; mean age 65.3 ± 6.1 years) participated in Experiment 2 (Reminder + Support group). At baseline, the two groups were comparable regarding age, physical independence, and daily activity levels, and there were no significant differences in average step counts or medication adherence between the groups before the intervention. The relationship between each participant and their avatar was not disclosed, and participants were selected such that the groups were composed of residents from different floors within the facility. This study was approved by the Ethics Committee of Gunma University Graduate School of Medicine (approval no. HS2024-273, approved April 2024). All study procedures were conducted after obtaining ethical approval. Data were collected between 6:00 and 20:00, and weekends were excluded to avoid excessive intervention fatigue. The overall study design is shown in Figure 1.

### 3.2 Standalone reminder App intervention

**Figure 2:**
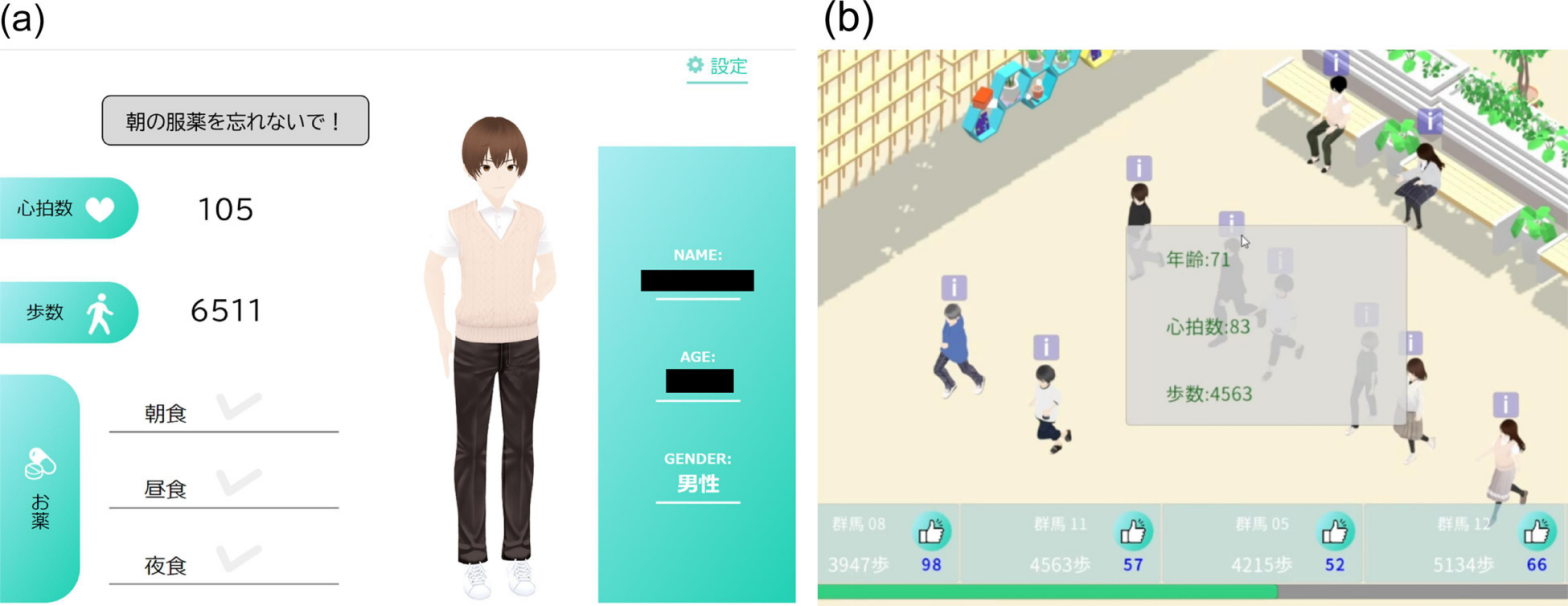
User interface of the intervention systems. **(a)** Reminder app showing medication notifications and daily step goal display. **(b)** Healthcare community app showing avatar activity status, interaction features (e.g., “Like” button), and visualization of other participants’ activity levels.

In Experiment 1 (Reminder group), participants received an intervention using our custom-built health management app with reminder functionality. During the four-week baseline period, participants wore a wrist-worn wearable device (Google Pixel Watch), provided by the research staff, at all times, which recorded their physical activity (step counts) and medication intake. However, no prompts or app notifications were provided during this period, and step counts and medication adherence were measured under normal living conditions. During the subsequent four-week intervention period, participants continued to wear the Pixel Watch with the reminder app enabled to send notifications. This app issued alarm notifications at preset medication times and displayed messages daily regarding the step goal, providing regular reminders aimed at promoting medication adherence and physical activity. Individual participants’ step counts and medication record data were transmitted to a cloud server and collected for research. However, no data sharing or social features were implemented in Experiment 1. Each participant only received notifications on their own device and did not exchange data or interact with other participants. During the intervention, the research staff assisted with device usage and responded to questions as needed, but participants were expected to self-manage their daily exercise and medication behaviors in response to the reminders. The system interfaces for Experiments 1 and 2 are shown in Figures 2a,b, respectively. Figure 2a illustrates the reminder-based interface, including medication notifications and daily step goal displays. Figure 2b presents the healthcare community interface, where avatar activity status, visualization of other participants’ behaviors, and interactive features such as the “Like” button are integrated. Key functional components are annotated in English within the figures to facilitate interpretation by international readers. All interface elements are presented with English annotations to facilitate global reproducibility, and the study consistently refers to participants as ‘older adults’ to align with inclusive medical standards.

### 3.3 Healthcare community App combined intervention

In Experiment 2 (Reminder + Support group), after the same four-week baseline observation period, an intervention was conducted that combined the app's reminder functionality with an avatar-sharing healthcare community system. The interventions used the “Health Care Community” application (V 1.6.1), developed on the Hyperledger Fabric platform for Android OS. As in Experiment 1, during the baseline period, only step counts and medication data were collected via the Pixel Watch, and no intervention notifications were provided. During the four-week intervention period that followed, participants continued to wear the Pixel Watch and used the “Health Care Community” application developed by the research team on a tablet. This system retained the reminder functions used in Experiment 1 (medication-time notifications and daily activity reminders) while featuring an interactive avatar-sharing function in a virtual space. Data collected from the Pixel Watch, such as each participant's total daily steps, exercise intensity, and medication timing, were sent to the cloud server and reflected on the participant's avatar in the virtual space. For example, an avatar with a small step count was displayed sitting on a bench, whereas an avatar with a high step count was shown running, thereby visually representing real-world health behaviors in a virtual environment. All 10 participants in Experiment 2 participated in the same virtual environment simultaneously, allowing them to view each other's avatars. Each participant's identity was protected by not disclosing any personal information beyond the avatar's name, enabling anonymous comparison and observation of activities. Additionally, to promote social support among participants, a feature was introduced that allowed each participant to anonymously “like” another participant's avatar (approval mark) once per day. The recipient received a notification of the “like” on the app. Moreover, the system anonymized all collected participant behavior data with privacy in mind and recorded them on a private blockchain based on Hyperledger Fabric. The introduction of blockchain technology was intended to prevent data tampering and ensure data integrity, such that once recorded, step counts and medication information could not be altered. However, given the small cohort size, individual identities may be vulnerable to re-identification via linkage attacks; therefore, future system iterations should integrate Zero-knowledge Proofs (ZKP) to satisfy the “zero-knowledge” requirement. This provides participants with the reassurance that their efforts are securely recorded and verified, and allows activity data to be reliably shared with third parties (such as care staff or researchers). Before the intervention, participants in Experiment 2 were instructed on how to operate the tablet and app, including how to log into the virtual space and view their avatars. In accordance with CONSORT-EHEALTH sub-item 5-x, we explicitly define the level of human involvement in this intervention. While the digital system provided automated reminders and avatar-based behavioral visualization, research staff were physically present for approximately 30 min per day to assist with technical troubleshooting, such as device charging, wearable synchronization, and tablet login support. Importantly, staff were strictly prohibited from providing verbal encouragement, behavioral coaching, or any form of motivational feedback during the intervention period. This restriction was implemented to ensure that any observed behavioral changes could be attributed primarily to the digital community features—such as avatar interaction and social feedback—rather than interpersonal influence from research personnel. Furthermore, data analysts were blinded to the group assignments during the entire statistical evaluation process to ensure the objectivity of the results. During the intervention, research staff provided technical support as needed and gave standardized procedural instructions on how to view other participants’ avatar activity levels and how to use the “Like” feature. Staff did not provide motivational encouragement or behavioral coaching beyond these technical instructions.

### 3.4 Evaluation items and data collection

In both experiments, the primary outcome measures were quantitative indices of physical activity and medication adherence, and the secondary outcome was subjective evaluation of the program. Baseline digital literacy scores (MDPQ-16) were also recorded to confirm participants’ ability to operate the system, although they were not treated as outcome variables. Physical activity was measured by daily step counts, with step data automatically recorded by the Pixel Watch accelerometer and aggregated daily. For medication adherence, we calculated the adherence rate (actual dose taken/prescribed dose) by comparing the number of medication intakes recorded by the app with each participant's prescribed daily dose count. Physical activity was measured by daily step counts, with step data automatically recorded by the Pixel Watch accelerometer and aggregated daily. For medication adherence, we calculated the adherence rate (actual dose taken/prescribed dose) by comparing the number of medication intakes recorded by the app with each participant's prescribed daily dose count. In Experiments 1 and 2, daily step counts and medication logs were collected during the intervention period and compared with the corresponding baseline data. In Experiment 2, as a secondary outcome, we also observed the amount of interaction in the virtual space by recording the total number of “Likes” each participant received. In accordance with CONSORT-EHEALTH recommendations on reporting intervention use, we defined use/engagement metrics as objectively captured interaction signals from the deployed platform. In the present pilot study, the usage-related indicators available for retrospective analysis were the frequency of successful health data synchronizations and the number of “Likes” received by each participant in the virtual space during the intervention period. In the present pilot study, the usage-related indicators available for retrospective analysis included the frequency of successful health data synchronizations, the number of “Likes” received by each participant in the virtual space, mean login frequency, and approximate daily time spent in the application. Specifically, participants logged into the system a mean of 3.4 times per day and spent approximately 5.0 ± 7.3 min per day in the application. In addition, the number of daily checks of the virtual space was confirmed to be up to three times per day, although a more granular distribution of checking frequency was not available for analysis. Accordingly, intervention “dosage” was primarily operationalized using the frequency of successful health data synchronizations and the total number of social interaction signals recorded per participant, while the other session-level usage indicators were treated as supplementary descriptive metrics. More detailed logfile-based measures will be prospectively collected in future trials using predefined operational definitions for session start, inactivity timeout, and visit frequency. These metrics will be prospectively logged in future trials using predefined operational definitions for session start, inactivity timeout, and visit frequency. At the end of the intervention, participants completed a questionnaire survey regarding their subjective program evaluation and perceived behavioral changes. Additionally, a subset of participants (*n* = 10 from each group, selected by voluntary participation) underwent brief semi-structured individual interviews with the research staff. Interviews followed a guide covering impressions of the program, perceived behavioral changes, facilitators and barriers to engagement, and the role of social features (Reminder + Support group only). Interview duration ranged from approximately 10 to 20 min. Free-text questionnaire responses and interview transcripts were analyzed using thematic analysis (25). Two researchers independently coded the data; disagreements were resolved through discussion until consensus was reached. Integration of quantitative and qualitative findings was achieved through a joint display approach (Table 4), in which key Likert-scale responses were aligned with representative participant quotes to identify convergent and divergent patterns. The effectiveness of the intervention was evaluated by integrating both quantitative and qualitative data.

For statistical analysis, paired tests within each group were conducted to compare the primary outcomes between the baseline and intervention periods. Specifically, considering the data distribution and sample size, we used the Wilcoxon signed-rank test to compare baseline values with each intervention week for both step count and medication adherence (significance level *α* = 0.05, two-tailed test). To account for multiple comparisons across the four intervention weeks, the significance threshold was adjusted using the Bonferroni correction (*α* = 0.0125). Results with *p*-values between 0.0125 and 0.05 were interpreted as suggestive trends rather than statistically significant findings. In addition, to directly compare the efficacy of the two interventions, a *post-hoc* between-group analysis was conducted using the Mann–Whitney *U* test on the change from baseline to Week 4.In addition, as an exploratory dose–response analysis within Experiment 2, we examined whether greater engagement in the virtual environment was associated with greater behavioral improvement. Specifically, Spearman's rank correlation coefficient was calculated between the total number of “Likes” received during the intervention period and the change in daily step count from baseline. For the questionnaire's quantitative items, mean Likert scores were calculated for each group, and a qualitative analysis was performed on the free-response answers and interview content to identify common themes and noteworthy comments. Data analysis was performed using Python and Matplotlib. Because this investigation was designed as a pilot feasibility study with a limited sample size (*N* = 10 per group), the analyses primarily aimed to explore behavioral trends and assess the practicality and acceptability of the intervention rather than to establish definitive causal efficacy.

## 4 Results

### 4.1 Quantitative assessment

**Figure 3:**
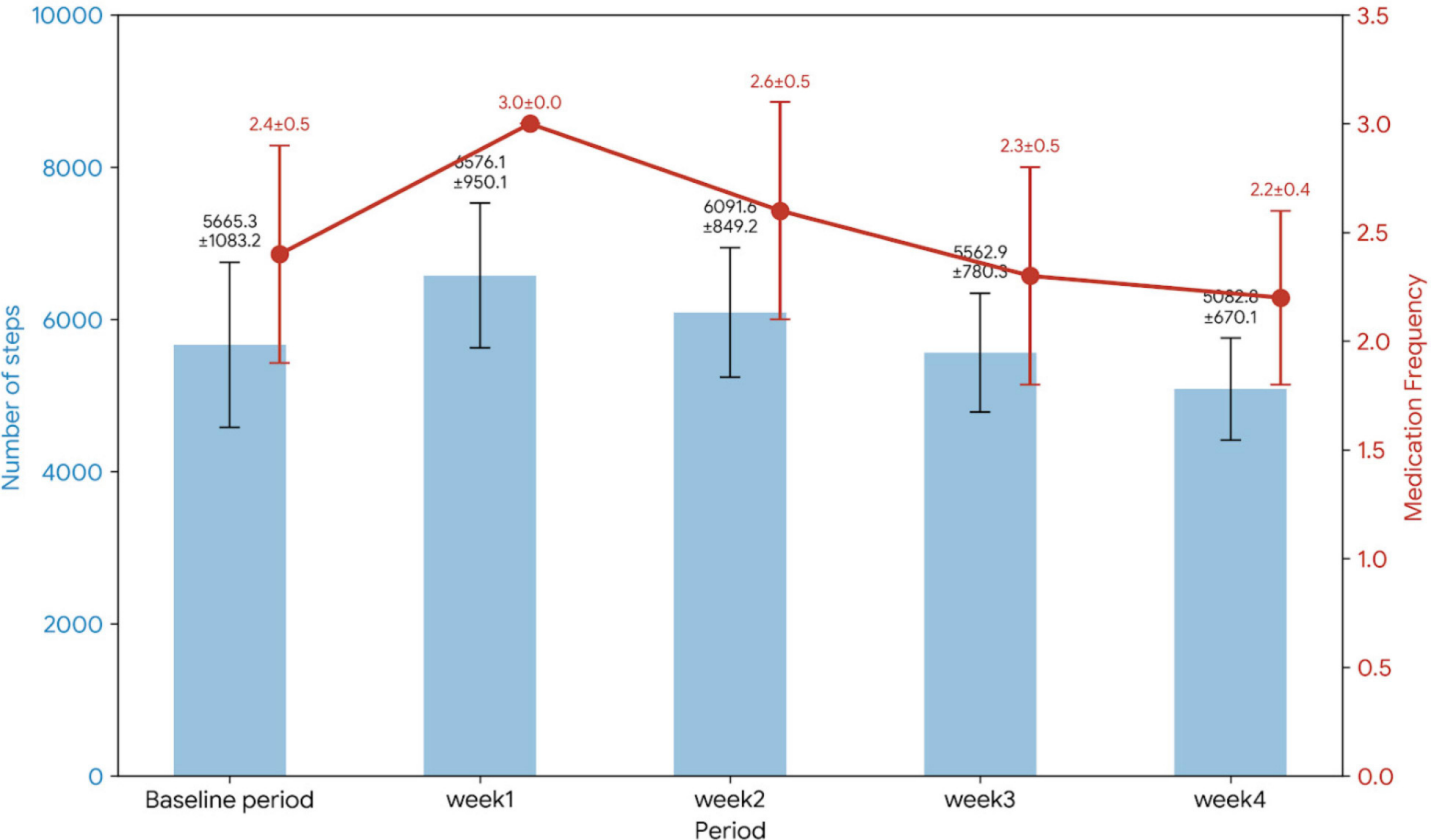
Reminder group results for changes in step count and medication adherence.

**Figure 4:**
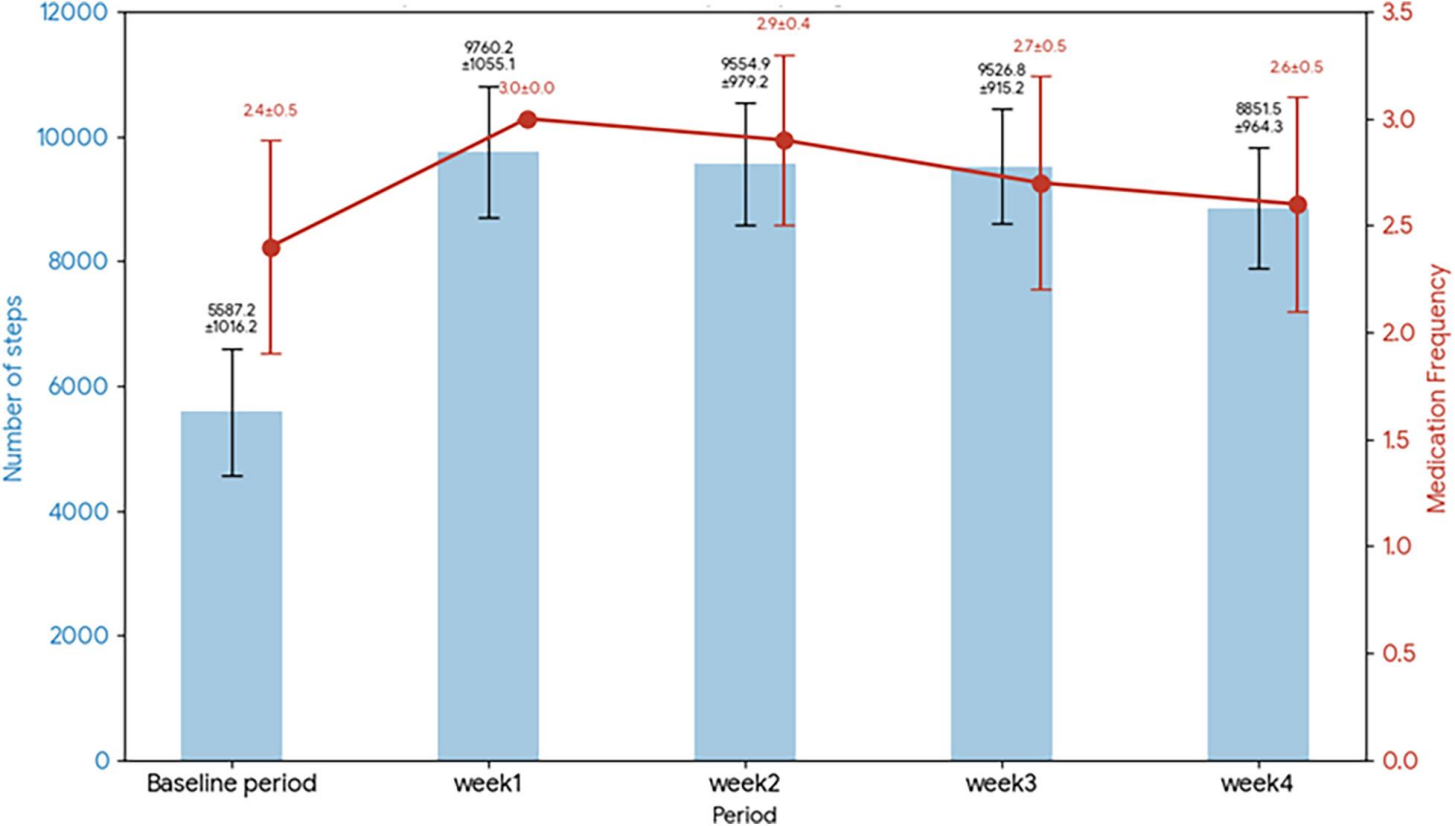
Reminder + support group results for changes in step count and medication adherence.

Because these two experiments were conducted as separate pilot studies with small samples (*N* = 10 per group), the analyses focus primarily on descriptive patterns and within-group pre-post comparisons. Any apparent differences between groups should therefore be interpreted cautiously. Figures 3, 4 show the results of Experiments 1 and 2 regarding changes in the primary outcome measures. Because both experiments were small pilot studies (*N* = 10 per group), the results are presented descriptively alongside within-group statistical tests. Table 1 reports the mean and standard deviation (SD) of step counts during each period, and Table 2 summarizes the statistical comparisons between each intervention week and the baseline period. To complement the *p*-value-based analysis, effect sizes (Cohen's r) were calculated for all Wilcoxon signed-rank tests. In the Reminder + Support group, the increase in step count during Week 1 exhibited a large effect size (*r* = 0.82), and large effects were generally maintained through Week 4, whereas the Reminder group showed attenuation to small effect sizes over time.

**Table 1:** Mean daily step counts and medication adherence across baseline and intervention periods.

Period	Week	Number of steps (Mean ± SD), (doses/day; percentage of prescribed doses, where 100% = 3 doses/day)		Medication adherence (Mean ± SD), (doses/day; percentage of prescribed doses, where 100% = 3 doses/day)	
		Reminder group	Reminder + Support group	Reminder group	Reminder + Support group
Baseline period		5,665.3 ± 1,083.2	5,587.2 ± 1,016.2	2.4 ± 0.5 (80%)	2.4 ± 0.5 (80%)
Intervention period	Week 1	6,576.1 ± 950.1	9,760.2 ± 1,055.1	3.0 ± 0 (100%)	3.0 ± 0 (100%)
	Week 2	6,091.6 ± 849.2	9,554.9 ± 979.2	2.6 ± 0.5 (87%)	2.9 ± 0.4 (96%)
	Week 3	5,562.9 ± 780.3	9,526.8 ± 915.2	2.3 ± 0.5 (77%)	2.7 ± 0.5 (90%)
	Week 4	5,082.8 ± 670.1	8,851.5 ± 964.3	2.2 ± 0.4 (73%)	2.6 ± 0.5 (87%)

Medication adherence is expressed as the mean number of doses taken per day. All participants in this study were prescribed a three-times-daily regimen; therefore, 3.0 doses/day corresponds to 100% adherence. The application interface imposed a hard ceiling of three recorded doses per day, which precluded detection of over-adherence and may have contributed to the lack of variance observed in Week 1 (3.0 ± 0.0 in both groups). This ceiling effect limits the sensitivity of the measure, particularly during periods of high adherence.

**Table 2:** Results of the Wilcoxon signed-rank test for the baseline and intervention periods, with effect sizes.

Intervention period	Number of steps (*p*-value/r)		Medication adherence (*p*-value/r)	
	Reminder group	Reminder + support group	Reminder group	Reminder + support group
Week 1	0.0,020/0.78	0.0,020/0.82	0.0156/0.55	0.0078/0.65
Week 2	0.0059/0.70	0.0020/0.80	0.2891/0.15	0.0156/0.55
Week 3	0.4316/0.05	0.0181/0.60	0.5000/0.03	0.0156/0.55
Week 4	0.3750/0.10	0.0039/0.75	0.1250/0.20	0.0313/0.50
Comparison	Outcome	Test	Statistic	*p*-value
Change baseline → Week4 (Reminder vs Reminder + Support)	Step count	Mann–Whitney *U*	11.0	*p*-value < 0.05

To adjust for multiple comparisons across four intervention weeks, the significance threshold was corrected using the Bonferroni method (*α* = 0.0125). *P*-values greater than 0.0125 are interpreted as non-significant trends, even if they are below 0.05.

In the Reminder group (Experiment 1), a temporary increase in both step count and medication intake was observed immediately after the start of the intervention; however, these effects did not persist. Following the application of the Bonferroni correction (adjusted *α* = 0.0125), several findings that were previously described as “suggestive” are now more appropriately interpreted as statistically non-significant trends. For example, the improvement in medication adherence observed in Week 2 of the Reminder + Support group (*p* = 0.0156) does not meet the corrected threshold for statistical significance. Table 2 has been updated accordingly to reflect these adjusted criteria, ensuring that all interpretations are conservative and minimizing the risk of Type I error inflation. The average daily step count during the baseline period was 5,665 steps, which increased by approximately 16% to 6,576 steps in Week 1 of the intervention. This increase was statistically significant (baseline vs. Week 1, *p* < 0.01). However, in Week 2 of the intervention, the average fell to 6,092 steps (a + 7.5% change from the baseline, *p* = 0.0059), then to 5,563 steps in Week 3 (−1.81%, *p* = 0.4316) and 5,083 steps in Week 4 (−10.3%, *p* = 0.3750), gradually declining. By Week 3 onward, the difference from the baseline was no longer significant. In other words, although an increase in steps was observed early in the intervention period, a tendency to return to the original level was observed thereafter. Both groups exhibited a downward drift in medication adherence following an initial peak in Week 1 (100%). In the Reminder group, the baseline mean medication intake was 2.4 doses per day (approximately 80% adherence). This increased to 3.0 ± 0.0 in Week 1 but gradually declined to 2.6 ± 0.5 in Week 2, 2.3 ± 0.5 in Week 3, and 2.2 ± 0.4 in Week 4, ultimately dropping below the baseline level. Conversely, the Reminder + Support group demonstrated greater resilience to this drift. Although adherence also peaked at 3.0 in Week 1, it remained relatively stable thereafter, reaching 2.6 ± 0.5 in Week 4, which remained numerically and statistically higher than its baseline value. The baseline average number of medication intakes per day (out of the prescribed dosage) was 2.4, which corresponded to approximately 80% adherence to a thrice-daily prescription. In Table 1, medication adherence is therefore presented as mean doses per day (± SD), where 3.0 doses/day represents full adherence to the prescribed regimen. In Week 1 of the intervention, the average rose to 3.0 per day (achieving 100% adherence as no doses were missed, *p* = 0.0156, indicating a suggestive but not statistically significant improvement under the Bonferroni-adjusted threshold), but it slightly decreased to 2.6 per day (approximately 87% adherence, *p* = 0.289) in Week 2, 2.3 per day (77%, *p* = 0.500) in Week 3, and 2.2 per day (73%, *p* = 0.125) in Week 4. The perfect uniformity observed in Week 1 medication data ($3.0 \pm 0.0$ for both groups) is attributed to a combination of high initial participant motivation and intensive staff guidance during the app-onboarding phase. Furthermore, the application interface imposed a hard ceiling of three recorded doses per day, which may have masked any variance among over-adherent participants. However, this lack of variance in the initial phase suggests a ceiling effect inherent in the application's tracking interface, which may have limited the detection of nuanced behavioral changes during the onboarding period. Therefore, the reminder-only intervention had only a transient effect, and by the end of the four-week intervention, there were no significant improvements in either step count or medication adherence compared with the baseline. The overall mean step count for the Reminder group throughout the 4-week intervention period was 5,828.4 steps/day, representing a nominal increase of 2.9% over the baseline period. This overall mean stands in stark contrast to the 16.1% improvement observed in the first week alone, highlighting the rapid attenuation of effect in the absence of social support.

By contrast, in the Reminder + Support group (Experiment 2), substantial improvements in behavioral indices relative to baseline were observed. The mean step count during the baseline period was 5,587 steps per day, whereas in Week 1 of the intervention, it increased to 9,760 steps (+74%, *p* < 0.01). A high level of walking was maintained throughout the intervention period. In Week 2, it averaged 9,555 steps (+71%, *p* < 0.01) and in Week 3, 9,527 steps (+70%, *p* < 0.01), remaining almost identical. Although the average step count declined slightly to 8,852 steps in the fourth week (+58%, *p* < 0.01), it still far exceeded the baseline level. The average step count over the entire intervention period (four weeks) was approximately 9,423 steps per day, representing an increase of over 60% compared with the baseline. For step counts, the intervention effect in the Reminder + Support group remained statistically significant (*p* < 0.01) at every weekly time point throughout the study period, although the magnitude of the improvement decreased from its peak of +74.7% in Week 1 to +58.4% by Week 4. Regarding medication adherence, the Reminder + Support group showed improvement relative to baseline throughout the study period, although only the Week 1 comparison reached statistical significance after Bonferroni correction. The average baseline medication intake was 2.4 per day (roughly 80% adherence). In Week 1 of the intervention, it reached 3.0 per day (100%, *p* < 0.01),remained relatively high thereafter: 2.9 per day in Week 2 (about 96%, *p* = 0.0156, indicating a non-significant trend under the Bonferroni-adjusted threshold), 2.7 per day in Week 3 (90%, *p* = 0.0156), and 2.6 per day in Week 4 (87%, *p* = 0.0313). Although these *p*-values were below 0.05, they did not meet the Bonferroni-corrected threshold of *α* = 0.0125, and are therefore interpreted as suggestive trends. Thus, in the Reminder + Support group, medication adherence remained numerically higher than baseline throughout the intervention. Although only Week 1 reached statistical significance after correction for multiple comparisons, the sustained numerical improvement suggests that the inclusion of avatar sharing and social engagement may have contributed to the maintenance of medication behavior.

In summary, a descriptive difference was observed between the two groups in the quantitative indicators of steps and medication. While the Reminder group showed some behavioral changes at the start of the intervention, the effect dissipated in a short time; however, the Reminder + Support group maintained improvement throughout the period, with step counts remaining statistically significant and medication adherence showing sustained numerical gains. In particular, at the end of Week 4 of the intervention, the Reminder + Support group had, on average, approximately 3,000 more steps per day than the Reminder group (8,852 vs. 5,083) and a medication adherence rate that was over ten percentage points higher (approximately 87% vs. 73%). To directly compare intervention efficacy, we performed a *post-hoc* between-group analysis on the change in step count from baseline to Week 4, which indicated a statistically significant difference in the change scores between the groups (Mann–Whitney *U* = 11.0, *p* < 0.05). These findings provide only preliminary and suggestive evidence that the Reminder + Support intervention may be more effective than the reminder-only intervention in promoting sustained physical activity. Because the study was based on small, non-randomized pilot groups, this between-group interpretation should be made cautiously and requires confirmation in adequately powered randomized controlled trials. As an exploratory engagement–outcome analysis in Experiment 2, social interaction within the virtual environment was quantified by the total number of “Likes” exchanged among participants. Over the four-week intervention period, participants received a mean of 89.4 likes (SD = 14.2). Other session-level usage metrics, such as login counts, app-use duration, and daily checks of the virtual space, were not consistently available in an analyzable retrospective format in this pilot dataset. We then examined whether greater engagement was associated with greater behavioral improvement. A Spearman's rank correlation analysis showed a significant positive association between the number of Likes received and the improvement in daily step count from baseline (rs = 0.65, *p* < 0.05), suggesting that participants who received more positive social feedback tended to show larger gains in physical activity.

### 4.2 Qualitative evaluation

A questionnaire survey (valid responses: 10 each in the Reminder and Reminder + Support groups) and participant interviews conducted after the intervention revealed differences in each group's subjective program evaluations and perceived effects. Regarding overall satisfaction, 90% (9/10) of participants in the Reminder + Support group responded that they were “satisfied with the program,” and 80% reported that they “enjoyed participating in it.” By contrast, in the Reminder group, only approximately 50% of participants answered that they were “satisfied” or “found it fun” (the remainder answered “neither agree nor disagree” or gave negative responses). Regarding self-assessed behavior change, 80% of the Reminder + Support group felt that they had “become more proactive in moving their bodies than before” and that their “awareness of properly taking medication had increased.” However, in the Reminder group, only approximately 30% reported that they had “developed a habit of physical activity” and 40% said their “awareness of medication had increased”; the rest of the participants self-assessed that “there was not much change” in their activity and medication behaviors. These differences in subjective evaluations are consistent with the trends in behavioral measures indicated by the quantitative results. Table 3 shows the survey results; however, five items that are unrelated to Experiment 1 have been removed.

**Table 3:** Survey results.

Questionnaire item	Experiment 1	Experiment 2
Q1. Did you compare your avatar's activity level with those of other participants?	–	4.8
Q2. Upon seeing other participants engaging in active exercise, did you strongly feel, “I should try harder too”?	–	4.5
Q3. Did you feel pressure or a sense of responsibility when you perceived that your avatar was not moving much?	–	4.5
Q4. Did you feel a sense of solidarity or companionship with other participants through activities in the virtual space?	–	5
Q5. Was receiving “Likes” from other participants a major source of encouragement for continuing exercise and medication adherence?	–	4.2
Q6. Do you feel confident that you can continue your exercise habits without using this system after the intervention period ends?	3.8	4.6
Q7. Do you feel motivated to continue using this system even after the intervention period ends?	3.5	4.5
Q8. Did using this system help you find exercise and medication adherence to be “fun”?	3.8	4.5
Q9. Was the operation of the tablet and wearable device easy?	4.9	4.5
Q10. Did you strongly feel that charging and maintaining the wearable device was a daily burden?	4.7	4.2
Q11. Did you sometimes feel annoyed by the notifications or reminders from the app or device?	4.5	4.4
Q12. Did you feel that this system (avatar) was effective in anonymizing your personal information?	3.1	4.8
Q13. Did you find that this system (avatar) was very useful for managing your personal health?	3.5	4.4
Q14. Did you understand the function of blockchain (data sharing)?	3	4.6
Q15. Did you have any resistance to your data being shared anonymously with other participants?	4.3	4.4

Items Q10 (burden of charging/maintenance), Q11 (annoyance with reminders), and Q15 (resistance to data sharing) are negatively worded; higher scores indicate greater perceived burden, annoyance, or resistance, respectively. These items were not reverse-coded, and their scores should be interpreted accordingly. The high scores on Q10 (4.2–4.7) and Q11 (4.4–4.5) indicate that participants in both groups experienced substantial burden and annoyance, representing important usability concerns.

When items related to social motivation were examined, strong positive responses were observed in the Reminder + Support group. Participants reported that they frequently compared their avatar's activity level with those of other participants (mean score 4.8 out of 5) and that seeing others exercising made them feel that they should try harder as well (4.5/5). Participants also reported feeling a sense of responsibility when their avatar appeared less active (4.5/5) and a strong sense of solidarity with other participants in the virtual space (5.0/5). In addition, receiving “Likes” from other participants was perceived as a significant source of encouragement (4.2/5), suggesting that social feedback contributed to maintaining motivation for continued exercise and medication adherence. These items were assessed only in the Reminder + Support group because the Reminder group did not have access to the virtual interaction features of the system.This suggests that the mild surveillance and cooperative environment mediated by anonymous avatars generated an appropriate level of tension and responsibility, which served as a driving force for continued behavior. Participants in the Reminder + Support group also indicated that receiving “likes” provided significant encouragement, suggesting that praise and empathy among participants contributed to maintaining motivation. Furthermore, for the question “Do you feel motivated to continue using this system even after the intervention period ends?” the Reminder + Support group gave strongly positive responses, averaging 4.5, whereas the Reminder group scored significantly lower at 3.5. By contrast, for the question “Do you feel confident that you can continue your exercise habits without using this system after the intervention period ends?,” the Reminder group scored 3.8 vs. 3.5 for the support group, indicating slightly higher confidence in the Reminder group. These results suggest that participants in the Reminder + Support group strongly felt the benefits of the system's support and had a high desire to continue using it after the intervention, whereas slightly more participants in the Reminder group believed that they could manage without the system. In the Reminder group, a participant remarked that “in the end, one has to rely on one's own willpower,” whereas, in the Reminder + Support group, a participant reported a sense of psychological support, expressing that “I was able to continue thanks to the system.”

Results for items related to technical acceptability were mixed. While ease of use was rated highly (Q9: 4.5–4.9), negatively worded items revealed substantial concerns: participants reported considerable burden from device charging and maintenance (Q10: 4.2–4.7) and notable annoyance with reminders (Q11: 4.4–4.5). Resistance to anonymous data sharing was also moderately high (Q15: 4.3–4.4). These findings indicate that although the system was technically operable, the frequency of notifications and daily maintenance requirements posed meaningful usability challenges. For example, in response to the question “Was the operation of the tablet and wearable device easy?,” both the Reminder and Reminder + Support groups gave high average ratings (4.9 and 4.5, respectively), suggesting that the user interface was generally usable without difficulty for older adults. However, some free-text responses indicated issues such as “Initially, I was confused about how to remember the login procedure” and “Sometimes I forgot to charge the watch,” highlighting challenges related to the initial learning curve of the digital devices and the burden of ongoing management (e.g., charging). Regarding the question “Did you sometimes feel annoyed by the notifications or reminders from the app or device?,” both groups had relatively high average scores (4.4–4.5, where 5 corresponds to “strongly felt”), indicating that some participants found the frequent reminder notifications annoying. Finally, with respect to the perceived effectiveness of the system's anonymity, the Reminder + Support group strongly agreed (4.8/5) with the statement “This system (avatar) effectively anonymized personal information and made me feel secure,” whereas the Reminder group gave a low score of 3.1. Participants in the Reminder + Support group felt that they could interact with others while being assured that their identity would be concealed by their avatar, an important psychological factor that was not present in Experiment 1. Additionally, the support group gave a high rating (4.4) to the question “Did you find that this system (avatar) was very useful for managing your personal health?” This suggests that the approach of connecting with others under guaranteed anonymity is helpful for self-directed health management.

Overall, the questionnaire and interview results indicated positive experiences in the Reminder + Support group, with specific comments including “The virtual connection with others acted as a stimulus and allowed me to continue enjoyably,” “I was able to exercise in a game-like manner, so even someone who dislikes exercise could keep going,” and “Since everyone was working on it together, I couldn’t slack off and felt a good sense of tension.” By contrast, in the Reminder group, there were comments such as “Even with notifications, in the end it's a battle with yourself,” suggesting the limitations of an intervention based solely on reminders. These subjective findings reinforce the quantitative results, indicating that the intervention incorporating shared avatars in a virtual space provided older participants with fresh enjoyment and social stimulation, thereby promoting positive behavioral change. To further integrate quantitative and qualitative findings, we constructed a joint display (Table 4) that aligns key Likert-scale responses with representative participant quotes. This approach enables a deeper understanding of the mechanisms underlying observed behavioral changes by linking numerical trends with experiential narratives.

**Table 4:** Joint display integrating quantitative feasibility ratings and qualitative interview data.

Domain	Quantitative finding（Likert）	Representative qualitative quote
Social comparison	Q1: 4.8/5	“I often checked others’ avatars and realized I was less active.”
Motivational contagion	Q2: 4.5/5	“Seeing others exercising made me feel I should move more.”
Responsibility/pressure	Q3: 4.5/5	“I didn't want my avatar to look inactive compared to others.”
Solidarity	Q4: 5.0/5	“Knowing my neighbors were also active made me get off the bench.”
Social reward	Q5: 4.2/5	“Receiving ‘likes’ made me feel recognized and encouraged.”
Sustained engagement	Q7: 4.5/5	“It felt like a game, so I wanted to keep going.”
Domain	Quantitative finding(Likert)	Representative qualitative quote

## 5 Discussion

This pilot feasibility study explored two approaches for supporting health behavior among residents of older adult care facilities: a reminder-only intervention and a healthcare community intervention incorporating avatar sharing. The results showed that the reminder-only approach produced only temporary increases in physical activity and medication adherence, which diminished over time. When the persistence of behavioral change was evaluated using the Week 4 endpoint, the Reminder group had declined to 5,082.8 steps per day (−10.3% relative to baseline), whereas the Reminder + Support group maintained 8,851.5 steps per day (+58.4% relative to baseline). Notably, a slight downward trend in step counts was observed in the Reminder + Support group by Week 4 (8,851.5 steps) compared to the initial peak in Week 1 (9,760.2 steps). Although this decline was not statistically significant, it suggests the presence of a potential “novelty effect,” whereby the initial enthusiasm and engagement with the avatar-based intervention may diminish over time. This phenomenon is commonly reported in digital health interventions, where early engagement is driven by curiosity and perceived novelty, followed by gradual habituation. To address this challenge, future implementations should incorporate adaptive gamification strategies, such as dynamic avatar evolution, personalized feedback loops, or periodic social events within the virtual environment, to sustain user engagement and mitigate behavioral decay over longer intervention periods. This contrast suggests that social engagement mechanisms may have contributed to the persistence of behavioral change, although this interpretation is limited by the non-randomized design and small sample size.

First, we compare the increase in physical activity observed in this study with that reported in the literature. When evaluated at the Week 4 endpoint, the Reminder + Support group still maintained a substantial increase in step count (+58.4%), whereas the Reminder group showed a decline below baseline (−10.3%). This descriptive contrast suggests that social engagement mechanisms may play a role in sustaining behavioral change, although the non-randomized design precludes causal inference. For example, Li et al. (2) reported an average increase of only approximately 10% in daily steps (standardized mean difference +0.28) in a meta-analysis of activity tracker interventions among older adults. By contrast, the approach used in our intervention, which integrates social elements into digital technology, has the potential to elicit much larger behavioral changes. Similarly, the Reminder + Support group's average medication adherence rate improved by **13.3 percentage points (from 80.0% to 93.3%)**. Generally, medication adherence interventions among older adults are considered to have a moderate effect (mean effect size g ≈ 0.5), and many studies report an improvement of only about 10 percentage points (4). Therefore, the 14-point increase observed in this study exceeded that of existing digital interventions. However, the Reminder group did not achieve significant long-term improvement in either step count or medication adherence, suggesting that simple reminder notifications alone are insufficient to fundamentally change habitual behaviors among older adults. Alert fatigue has been identified as a challenge in digital health interventions for older adults (8), and the phenomenon seen in the Reminder group—namely, a diminishing response to notifications over time—can be considered a typical example of this fatigue effect. The observed decline to 5,082.8 steps in Week 4 (−10.3% below baseline) in the Reminder group indicates that simple notification-based strategies may not only lose efficacy but could potentially lead to behaviors lower than baseline levels, underscoring the risk of “alert fatigue” and its potential to diminish intrinsic motivation. In contrast, the Reminder + Support group demonstrated resilience against this decay, suggesting that social cues mediated by avatars can transform an externally-driven prompt into an identity-based behavior, thereby mitigating the effects of psychological reactance. This phenomenon may be attributed to psychological reactance, where the frequent alerts are perceived as a threat to the participants’ autonomy, or “app fatigue” resulting from a cumulative cognitive burden. Based on these comparisons, our preliminary results suggest that the community-sharing intervention used in this study may help address some limitations of traditional interventions that rely solely on digital technology, although confirmation in larger randomized trials is needed.

Next, we consider the background factors that led to differences in the effects of the two interventions. A distinctive feature of this study was that participants could loosely monitor each other's activities through avatar sharing in a virtual space. Participants in the Reminder + Support group made comments such as “I felt that my avatar wasn't moving as much as others, so I decided to walk a bit more,” and “Since I was exercising in front of others, I couldn't slack off.” This suggests that an appropriate sense of tension and responsibility arises from being aware of others' presence, which in turn serves as a driving force for maintaining behavior. From a theoretical perspective, the effectiveness of the Shared Avatar system can be fundamentally explained through Bandura's Social Learning Theory, particularly the mechanism of self-efficacy formation through observational learning and modeling (11). In this framework, participants observe the behaviors of others and adjust their own actions based on perceived outcomes and social feedback. In the present intervention, seeing a peer's avatar actively moving within the virtual space functioned as a form of vicarious experience, demonstrating that the target behavior (e.g., walking or adhering to medication) was both achievable and normatively expected. Furthermore, the “Like” feature served as a form of vicarious reinforcement, providing visible social rewards that signaled approval and value for engaging in health-promoting behaviors. These mechanisms likely enhanced participants’ self-efficacy, defined as the belief in one's ability to execute specific actions required to achieve desired outcomes. By repeatedly observing successful behaviors and associated social rewards in the virtual environment, participants may have internalized both the feasibility and desirability of these behaviors. This process bridges the gap between knowledge (e.g., knowing that exercise is beneficial) and actual behavioral execution, transforming passive awareness into active self-regulation. Importantly, the system's design—particularly its use of anonymity—may have reduced social anxiety and fear of negative evaluation, allowing participants to engage more freely in observational learning and social comparison. Taken together, the integration of avatars, social feedback (“Likes”), and anonymity can be interpreted as a digitally mediated implementation of Social Learning Theory, which provides a coherent psychological explanation for why the Reminder + Support group demonstrated more sustained behavioral change than the Reminder-only group. This theoretical interpretation is further supported by the joint display analysis, which demonstrates that subjective experiences of social comparison, solidarity, and reward correspond closely with observed behavioral improvements. These mechanisms may be further amplified in future systems that adopt more immersive avatar representations, as stronger embodiment and social presence could deepen users’ sense of co-presence and behavioral engagement. Furthermore, we cannot overlook the fact that a sense of camaraderie and solidarity was fostered within the virtual community to which all participants belonged. In the Reminder + Support group, many participants reported feeling “the novelty and enjoyment of connecting with others in the virtual space” and “encouraged by knowing that others are working hard in the same space,” suggesting that positive social emotions such as reduced feelings of loneliness and awakened consciousness of cooperation may have facilitated behavioral change. The community intervention in this study was not limited to technical support, and its ability to engage with participants’ psychological and social aspects is essential for achieving the strong effects observed.

Furthermore, the design of this intervention promoted a sense of security through anonymity, thereby contributing to its effectiveness. While the proposed system utilizes a private blockchain (Hyperledger Fabric) to ensure data integrity, the small size of the cohort (*N* = 10) introduces inherent privacy challenges. Specifically, individual identities may be vulnerable to re-identification through linkage attacks if daily activity patterns (e.g., specific medication times or step count fluctuations) are correlated with observable real-world behaviors in the facility. Although avatars provide a level of pseudonymization, they do not fully eliminate the risk of inference attacks in tightly knit environments. Future research should explore the integration of advanced privacy-preserving techniques, such as differential privacy or zero-knowledge proofs (ZKP), to enhance data protection. In particular, ZKP could enable verification of behavioral compliance without revealing underlying personal data, thereby satisfying the “zero-knowledge” requirement in which the verifier learns nothing beyond the validity of the statement. Older adults may be reluctant to have their personal health data or lifestyle known to others; however, in this system, information sharing occurred via avatars in a virtual space, preventing individual identification. According to the questionnaire results, participants in the Reminder + Support group strongly felt that “anonymization by avatars was effective in protecting privacy” and gave them a sense of security, which likely lowered psychological barriers to both competition and cooperation, enabling greater engagement in the virtual space. Conversely, in the Reminder group, data were not shared with others, so participants experienced neither privacy concerns nor the moderate sense of “being watched” that can promote motivation. Because sharing health information can be sensitive for older adults in real-world contexts, the anonymous group-participation mechanism demonstrated in this study could serve as a useful model for creating environments in which older adults feel safe while encouraging one another.

Another notable element in the Reminder + Support group was the mutual recognition among participants via sending and receiving positive feedback through anonymous “likes.” Many participants reported that receiving such social rewards encouraged them daily. This suggests that the reinforcing effect of social praise contributes to continued behavior. Previous research has also identified that praise and support from family and friends are factors that promote healthy behaviors among older adults (26). Despite the lack of direct face-to-face contact in this study, the mechanism of recognizing and praising each other's efforts through avatars visualized in the virtual space provided a form of pseudo-social support. This allowed participants to feel a sense of solidarity and monitoring, which likely contributed to the maintenance and enhancement of motivation.

By contrast, in the Reminder group, in addition to alert fatigue, a lack of intrinsic motivation may have contributed to the intervention's effects. While the reminder notifications initially served to make participants “realize [they] had forgotten,” some participants also noted “in the end, whether I did it or not was up to me, so it didn't last long.” This suggests that extrinsic motivational strategies alone are insufficient for sustaining behavioral change, and that long-term habit formation requires intrinsic motivation such as personal enjoyment or a sense of meaning. In our study, the Reminder + Support group's intrinsic motivation appeared to be strengthened by the enjoyment associated with avatar sharing and gamification, whereas the Reminder group lacked these elements. This may explain why their improvements plateaued after the first few weeks.

Taken together, the findings suggest that the potentially greater effectiveness of the healthcare community app, relative to the reminder-only intervention, may be related to a combination of psychosocial factors, including social comparison, anonymity, social recognition, and intrinsic enjoyment. These findings may also be interpreted within a broader translational trajectory of immersive technologies for older adults. Prior AR/VR research has suggested that, when appropriately designed for older users, these technologies may support physical, cognitive, psychological, and social engagement. From this perspective, the present 2D avatar-based approach may be viewed as a low-barrier and scalable step within a broader continuum of technology-mediated behavior support, especially in settings where more immersive hardware is not yet practical. Although the present intervention used a comparatively simple 2D interface with basic avatars, the observed behavioral and motivational effects suggest that even low-barrier virtual social representations can be meaningful in older adult care settings. At the same time, future extensions using more immersive modalities—such as 3D avatars, augmented reality (AR), or mixed reality (MR)—may further enhance behavior change by strengthening embodiment and social presence. In particular, a stronger sense of being with others in a mediated environment, together with more behaviorally expressive avatars, may intensify social comparison, mutual awareness, and encouragement, thereby reinforcing the mechanisms identified in the present study. Accordingly, the current 2D system may be understood as a pragmatic and scalable first step, especially in care settings where head-mounted or highly immersive systems are not yet practical. The joint display analysis suggests that avatar-mediated social mechanisms—such as comparison, solidarity, and social reward—functioned as key drivers of sustained behavioral change. Each of these factors plays an important role in supporting behavioral change among older adults, and this study suggests that enabling these mechanisms through a digital platform may contribute to better outcomes than reminder-only approaches, although this possibility should be tested in larger confirmatory studies.

Despite its important contributions, this study has several limitations. A primary limitation of this study is its pilot feasibility design and small sample size (*N* = 10 per group), which were constrained by the capacity of the participating residential facilities. Accordingly, the study was not adequately powered to provide definitive estimates of intervention efficacy, detect subtle between-group differences, or support broad generalizability. In addition, the four-week intervention period limits inference regarding longer-term maintenance of behavior change. Therefore, the present findings should be interpreted as preliminary and hypothesis-generating rather than confirmatory. Future studies should evaluate this intervention in larger, adequately powered, randomized, multi-site trials. In addition, participants were required to meet a minimum level of digital literacy (MDPQ-16 ≥ 20), which may limit the applicability of the findings to older adults with lower digital proficiency. Another limitation concerns the granularity of logfile-based usage data in this pilot deployment. Although summary-level indicators such as mean login frequency (3.4 times/day), approximate daily time spent in the application (5.0 ± 7.3 min/day), and a maximum of three daily checks of the virtual space were available, more detailed session-level logs were not consistently retained in a format suitable for comprehensive behavioral analysis. Therefore, our assessment of intervention use and engagement still relied primarily on the number of “Likes” exchanged and health data synchronization frequency as objective indicators, rather than on a complete set of time-stamped exposure metrics. Future trials should prospectively collect detailed usage logs using predefined operational definitions for session start, inactivity timeout, and visit frequency. Furthermore, the measurement of medication adherence in this study was constrained by a ceiling effect inherent in the application design. The app interface imposed a hard ceiling of three recorded doses per day, meaning that any intake beyond this limit could not be captured. Because all participants were prescribed a three-times-daily regimen, the maximum recordable value (3.0 doses/day) coincided with 100% adherence, leaving no room for variance among fully adherent participants. This is reflected in the Week 1 data, where both groups achieved 3.0 ± 0.0 doses/day—a value that may represent a measurement artifact rather than genuinely uniform perfect adherence. The combination of high initial motivation during the onboarding phase and intensive staff guidance with device setup likely contributed to this uniformity, but the hard ceiling precluded any differentiation among participants who may have differed in their actual adherence patterns. This constraint reduces the sensitivity of the adherence measure, particularly during periods of high compliance, and may have attenuated detectable between-group differences. Future studies should adopt adherence measures with greater discriminative capacity, such as Medication Event Monitoring System (MEMS) caps that record exact dosing timestamps, proportion-of-days-covered (PDC) calculations derived from pharmacy dispensing records, or self-report instruments validated for older adult populations (e.g., the Morisky Medication Adherence Scale). In addition, if an app-based recording method is retained, the interface should be redesigned to allow recording of each individual dose event without an artificial daily cap, thereby enabling more granular analysis of adherence trajectories. Second, because this study was conducted in a specific environment (older adult care facilities), it is unclear whether similar effects would be observed in other populations such as older adults living at home. The way social motivation is perceived may differ depending on the characteristics of the target population. Therefore, future studies should investigate the applicability of this approach across diverse living environments. Third, several technology- and usability-related issues were noted. Although research staff provided support as needed during the intervention, practical implementation will require more intuitive user interfaces and support systems so that caregivers or family members are not overburdened (22). Participant feedback such as “I was confused by the login method at first” and “I tend to forget to charge the device” underscores the importance of designing devices that are easy for older adults, especially those unfamiliar with digital devices, to use. Possible improvements include automatic login functions, enhanced charging reminders, and voice assistance support. Despite the generally high ratings for ease of use, the intervention was not entirely free of friction. Questionnaire responses indicated relatively high levels of annoyance related to repeated reminders (mean score approximately 4.4–4.5 out of 5.0) and a perceived burden associated with daily system maintenance tasks such as device handling and charging (mean score approximately 4.2–4.7). These findings suggest that although the system was technically operable within the care facility environment, the frequency of notifications may have approached a threshold of alert fatigue. Excessive reminders could reduce user satisfaction and potentially undermine the long-term sustainability of mHealth interventions. Future implementations should therefore consider adaptive reminder strategies, such as personalized notification frequency or context-aware prompts, to balance motivational support with user burden. In addition, if future versions adopt more immersive modalities such as VR-, AR-, or MR-based interfaces, careful attention will be needed regarding device burden, staff support requirements, implementation constraints in care facilities, and possible adverse effects such as cybersickness or sensory overload in older adults. Fourth, there is room for improvement in the reward design. This study relied on social recognition rather than financial incentives to motivate behavior; however, future systems might consider issuing tokens (points) based on healthy behaviors using blockchain technology and visualizing them as rewards (23). This approach aligns with the recently proposed concept of a “token economy” for health behaviors, potentially enhancing motivation without increasing ethical or financial burdens. By addressing these issues and exploring these improvements, we aim to fully realize the potential of the older adult support app demonstrated in this study and further develop it into a more sustainable and versatile intervention program.

## 6 Conclusion

In this study, we introduced a healthcare community app that combines reminder notifications with virtual space avatar sharing in an older adult care facility and provided preliminary evidence suggesting the potential effectiveness. Improvements in step count and medication adherence were limited and transient in the reminder-only intervention but sustained in the avatar-sharing community intervention, suggesting that this approach is a potentially promising digital strategy for promoting health behavior change among older adults, pending confirmation in adequately powered randomized trials. This intervention incorporated the unique strengths of loose social connections among participants and gamification elements not found in conventional individually targeted support applications. In particular, elements such as being able to interact with and motivate peers while maintaining anonymity played important roles in promoting older adults’ willingness to participate without resistance. These findings underscore the significance of integrating technology and social factors into digital health interventions for older adults and propose a new direction for healthcare support in a super-aged society.

The healthcare community app developed and applied in this study has wide applicability in practical settings for older adult support. For example, if this system is implemented in group care environments such as nursing facilities or daycare centers, programs could be designed that allow users to motivate one another while engaging in rehabilitation exercises or managing medication. Even in settings where care staff traditionally provide individualized prompts and management, the app enables participants to independently increase their activity levels and adhere to medication schedules in a gamified manner, potentially reducing caregiver burden and revitalizing group rehabilitation. Moreover, when implementing this app intervention with older adults living at home, it would be possible to share and praise health efforts in a virtual space, even across geographical distances, thereby creating a social health support network that extends beyond local regions and families. By extending the data-sharing functionality with blockchain, family members and healthcare providers could securely access activity data and provide feedback, offering a comprehensive platform for older adult care that connects homes, care facilities, and medical institutions.

This study suggests that promoting health behavior change among older adults requires a comprehensive support design that incorporates social elements and psychological reassurance, rather than relying on a single technological intervention. Future digital health designs should actively create environments in which users can influence each other and sustain participation in an enjoyable manner, rather than simply providing reminders or recording functions. In doing so, it is important to address considerations unique to older adults—including privacy protection and ease of use—and the findings of this study provide concrete guidance for achieving this balance. By integrating digital technology and social interaction, such approaches may have the potential to support older adults’ exercise and medication habits and contribute to the extension of healthy life expectancy. The results of this pilot study represent a preliminary step toward developing next-generation older adult healthcare support models and highlight the need for larger confirmatory trials and consideration of practical implementation strategies.

## Data Availability

The datasets generated and analyzed during this study are not publicly available due to privacy and ethical restrictions pertaining to participant health data, but are available from the corresponding author on reasonable request.
